# Constructing origami power generator from one piece of electret thin film and application in AI-enabled transmission line vibration monitoring

**DOI:** 10.1038/s41378-023-00572-6

**Published:** 2023-08-07

**Authors:** Boming Lyu, Huipeng Zhou, Yangyang Gao, Xinhui Mao, Fangzhi Li, Jiyuan Zhang, Dezhi Nie, Wen Zeng, Yonglin Lu, Jin Wu, Zhaoshu Yang, Kai Tao

**Affiliations:** 1 Ministry of Education Key Laboratory of Micro and Nano Systems for Aerospace, Northwestern Polytechnical University, Xi’an, PR China; 2 Research & Development Institute of Northwestern Polytechnical University in Shenzhen, Shenzhen, PR China; 3 Research Institute of State Grid Jiangsu Electric Power Co., Ltd, Nanjing, PR China; 4 State Key Laboratory of Optoelectronic Materials and Technologies and the Guangdong Province Key Laboratory of Display Material and Technology, School of Electronics and Information Technology, Sun Yat-sen University, Guangzhou, PR China; 5 National Key Laboratory of Human Factors Engineering, China Astronaut Research and Training Center, Beijing, PR China

**Keywords:** Nanosensors, Electrical and electronic engineering

## Abstract

One of the crucial issues for applying electret/triboelectric power generators in the Internet of Things (IoT) is to take full advantage of specific high voltage signals and enable self-powered sensing. Therefore, inspired by Miura-origami, we present an innovative origami power generator (OPG) constructed from only one piece of electret thin film. The Miura-origami architecture realizes a generator with excellent deformability and stretchability and makes it unnecessary for any auxiliary support structure during the compress-release cycle. Various parameters of the generator are intensively investigated, including the excitation accelerations, excitation displacements, numbers of power generation units and deformation degree of the device. When stimulated with 5.0 g acceleration at 15 Hz frequency, the generator with 8 generation units can obtain an instantaneous peak-to-peak voltage and a remarkable optimum peak power of 328 V and 2152 μW at 50 MΩ, respectively. In addition, the regulable shape and multiple generation modes of the device greatly improve its applicability in various vibration energy collection requirements. Based on the above results, a hexagonal electret generator integrated with six-phase OPGs is developed as a “Buoy on Sky,” after which the signal waveforms generated from internal power generators are recognized with 92% accuracy through a neural network algorithm that identifies the vibration conditions of transmission lines. This work demonstrates that a fusion of origami art and energy conversion techniques can achieve a multifunctional generator design satisfying the requirements for IoT applications.

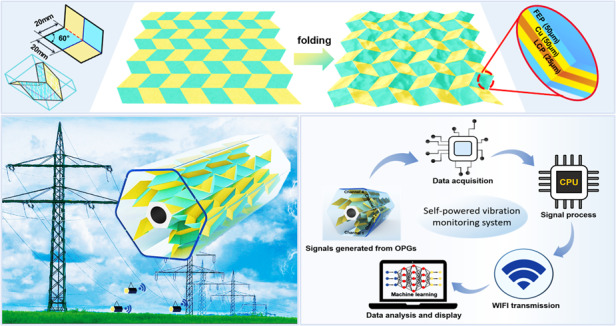

## Introduction

With the rapid development of the Internet of Things and portable electronics, various sensors have been applied in fields such as human-computer interaction, health care, and environmental monitoring^1–4^. In power grid, conductors and arresters in overhead transmission lines are constantly affected by wind, ice, low temperature, and other adverse weather conditions; these lines are thus prone to intense vibration that seriously threatens the security and stability of grid systems^5^. Therefore, many governments and companies have been conscious of constructing a distributed sensor network for secure prewarning of transmission line risks^5–8^. However, power supply is always a limitation in such large-scale distributed sensor network applications, as conventional batteries need to be replaced frequently. Thus, capturing clean energy from the natural environment and powering electronics is a practical solution^9–11^. At present, light energy^12^, mechanical energy^13,14^, thermal energy^15^, wave energy^16–18^, and acoustic energy^19^ are the predominant clean energy sources, among which mechanical energy has attracted much attention due to its wide availability and easy conversion. Mechanical energy can generally be converted into electrical energy by electrostatic^20–23^, electromagnetic^24–27^, piezoelectric^28–31^, and triboelectric methods^32–35^. In the field of low-frequency vibration energy harvesting (VEH), triboelectric nanogenerators (TENGs) based on contact electrification and electrostatic induction coupling have been proven to demonstrate excellent output performance^36–39^. In addition, its prominent features, such as the diverse selection of materials, simple structure, and easy processing^40–42^, provide great potential for satisfying the requirements of various energy harvesting scenarios.

Origami is an ancient art that has been documented for over 1,000 years. It can rely on the paper itself to realize ingenious structures or patterns. Origami-inspired structures have the characteristics of light weight, excellent flexibility, and deformability. Therefore, taking advantage of origami art in TENG design has been widely considered and investigated^43–46^. Some researchers have proposed origami-inspired TENGs with different structures, such as the zigzag-shaped TENG^45^ with a 7 g ultralightweight and the diamond-shaped TENG^46^ integrated with a supercapacitor. In our previous work, Kai Tao et al.^47^ further proposed an origami-inspired electret-based power generator on a liquid crystal polymer (LCP) substrate with a double helical spring structure. The compact origami structure made the device suitable for wearable devices and wave energy collection scenarios. However, none of the previous studies attempted to show origami-inspired power generators that could be applied to the IoT and self-powered vibration monitoring systems. Hence, we present a novel origami power generator (OPG) constructed from only one piece of multilayered electret thin film and explore its in-depth applications in the field of smart grids. The device presented in this work exhibits the following advantages:

- Unlike traditional multibody power generators, the OPG can be prepared from a single piece of electret thin film and return to its original state without auxiliary supporting structures.
- The OPG can be transformed into three generation modes by folding the original thin film into different structures, among which the Miura-origami structure can achieve the maximum capacitance variations in minimum space.
- The OPG can collect the vibrational energy from all angles and regulate its shape by modulating the arrangement of horizontal and vertical generation units, which is more conducive to collecting vibration energy in various scenarios.

In this work, we realized power generators based on vibration energy harvesting that can satisfy the demands of IoT applications to yield a self-powered monitoring system for the vibration recognition of transmission lines. First, based on the Miura-origami fold, we developed and fabricated a one-piece power generator with the characteristics of high stretchability, light weight, and self-recovery. Second, the output performances and characteristics of the generator were systematically measured. Then, the hexagonal electret generator (HEG) integrated with six-phase OPGs was developed as “Buoy on Sky” and rigidly suspended on the simulated transmission line platform. Furthermore, a vibration sensing system and an information exchange system of transmission lines were constructed based on the high-powered HEG. Finally, various vibration conditions were simulated, and the signal waveforms were collected from the six-phase OPGs, whereupon the signals were analyzed and recognized by neural network algorithms. The outcomes of our work prove the high effectiveness of the innovative electret power generator and demonstrate a novel application in the fields of VEH and IoT.

## Results and discussion

Miura-origami, invented by Japanese astrophysicist Koryo Miura, is regarded as one of the top 100 Japanese inventions and is widely used in satellites, solar panels, and soundproof walls. It allows flat materials with a large surface area to be transformed into simplified and compressed complex 3D structures. In the following sections, a one-piece origami power generator is developed based on Miura-origami and investigated by optimizing various parameters. Moreover, the HEG integrated with the six-phase OPGs is developed, characterized, and employed for recognizing the different vibration conditions of transmission lines.

### Device configuration

**Fig. 1:**
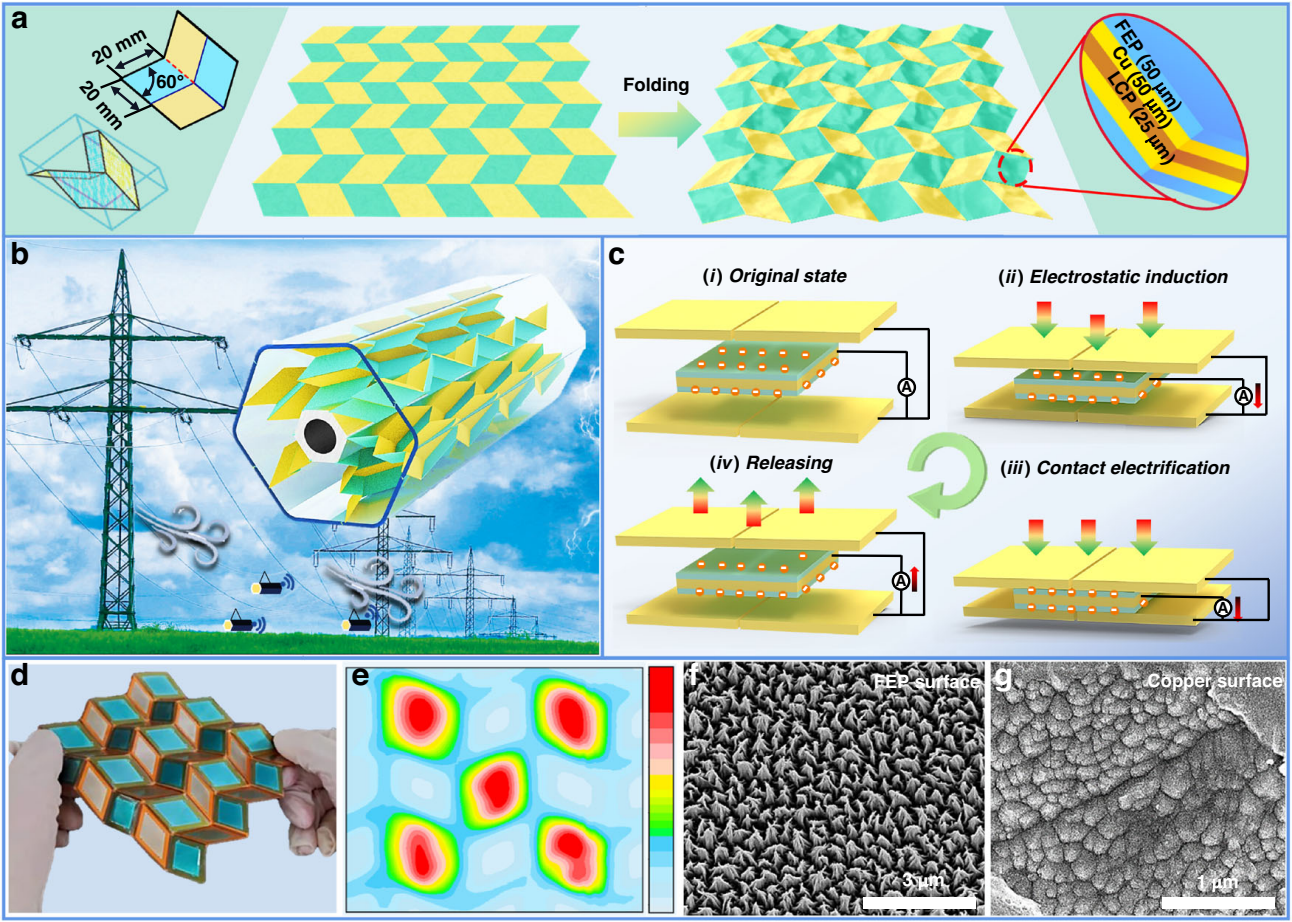
Configuration and formation of the proposed OPG device. **a** Schematic diagram of the Miura-patterned printed circuit board (PCB) and the fluorinated ethylene propylene (FEP) thin films connected in every other zigzag path; **b** HEG integrating six-phase OPGs with the function of monitoring the vibration conditions of transmission lines; **c** charge circulation in one compress-release cycle: (i) initial state; (ii) electrostatic induction when electrets and copper electrodes move forward with each other; (iii) contact electrification when the copper and FEP come into contact with each other; (iv) charge flows back when the device rebounds back to its initial state; **d** photograph of the OPG with 3 × 3 generation units; **e** charge distribution map of the Miura-patterned PCB board; **f** SEM image of the IBE-treated FEP surface; **g** SEM image of the dip-etched copper surface

Figure 1a shows a schematic diagram of the Miura-patterned PCB board with an array of parallelograms. The yellow areas represent the copper/LCP/copper composite substrates with thicknesses of 50 μm/25 μm/50 μm, and the green areas represent the FEP/copper/LCP/copper/FEP multilayered substrates with thicknesses of 50 μm/50 μm/25 μm/50 μm/50 μm. All parallelogram copper bases shown on the diagonal position are connected with each other, and the FEP electret films are attached to the PCB board in a zigzag path. Based on the folding rule of the single-generation unit, the flat materials are folded with alternating mountain folds and valley folds along the junction of the yellow and green areas. Then, the flat PCB boards can be packed into a compact shape by pressing the two ends together and unpacked by pulling on its opposite ends.

Due to the security issues with transmission lines in atrocious weather conditions, it is vitally important to construct a low-power consumption vibration monitoring system for a high-voltage transmission line system (HVTLS). To solve this problem, a hexagonal electret power generator integrated with six-phase strip-shaped OPGs (2 × 4 generation units) is presented here, which can serve the function of monitoring the vibration conditions of transmission lines. Figure 1b shows the schematic diagram of the highly integrated HEG suspended on the transmission lines.

Figure 1c shows the charge circulation of the OPG in one compress-release cycle. At the initial state, the conductive electrodes and the electrets are interleaved vertically, with one electret layer facing two conductive electrode layers simultaneously (i). Electrostatic induction occurs between the electrodes and electrets when the OPG is compressed with external pressure. The charge circulation mainly depends on the pre-implanted charge on the FEP surface (ii). Contact electrification occurs when the electrodes and electrets are intimately contacted (iii). The OPG returns to its original state after the external force is removed, resulting in the charge flowing in the opposite direction (iv). During the compress-release cycle, electrostatic induction and contact electrification coupling play crucial roles in maintaining the synchronous charge transfer mechanism.

Figure 1d shows a photograph of the OPG with 3 × 3 generation units. The blue films attached to the PCB boards in a zigzag path are the FEP electrets, and the electret thin film is folded along the junction of the electret areas and electrode areas. Figure 1e shows the Miura-patterned potential distribution map measured with the 3D potential scanner. After the corona discharge process, the entire surface is divided into “blue” and “red” areas, representing the neutrally and the negatively charged areas, respectively. The FEP areas can obtain a maximum surface potential of −2320 V, and the surface potential of the electrode areas is maintained at approximately 0 V. To promote the contact electrification mechanism within the FEP-Copper interfaces, the FEP electrets and copper surface are processed with ion beam etching (IBE) and dip-etching, respectively. Figure S1 shows the output voltages of the OPGs with different FEP surface microstructures. The OPG with IBE-etched FEP can achieve higher output performance than the OPG with the original FEP. Figure 1f, g shows scanning electron microscopy (SEM) images of the FEP electrets and copper surfaces, respectively.

### Output performance with different generation modes

**Fig. 2:**
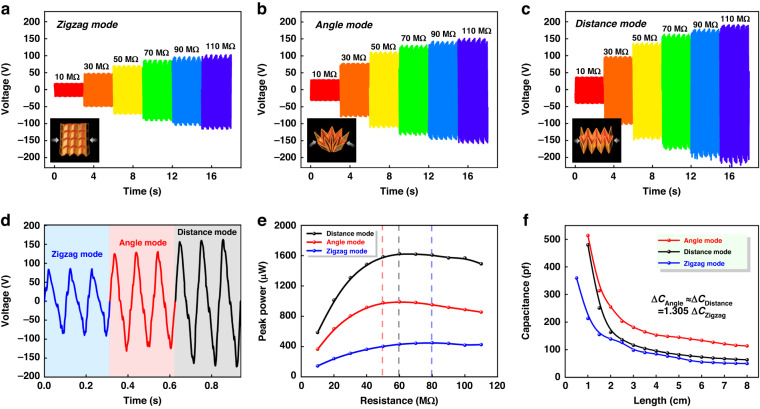
Output performance of the OPG with three generation modes. **a** Output voltages of the OPG in zigzag mode versus load resistance ranging from 10 MΩ to 110 MΩ; **b** output voltages of the OPG in angle mode versus load resistance ranging from 10 MΩ to 110 MΩ; **c** output voltages of the OPG in distance mode versus load resistance ranging from 10 MΩ to 110 MΩ; **d** output voltage comparison of the OPGs in three generation modes under 50 MΩ; **e** output powers of the OPGs in different generation modes versus load resistance ranging from 10 MΩ to 110 MΩ; **f** capacitance variation versus device thickness

The original Miura-patterned PCB boards are transformed into OPGs with three different generation modes. In zigzag mode, the original electret thin film is folded into a zigzag-shaped structure, and the FEP films are alternately placed. In the angle mode and distance mode, the original electret thin films are all folded into a Miura-origami structure, and the devices reciprocate the deformation in the circular and vertical directions, respectively. The optical images of the OPGs in the zigzag, angle, and distance modes are shown in Fig. 2a–c, respectively.

To demonstrate the advantages of the Miura structures in power generation, the capacitance variations of the zigzag structure and Miura structure in one compress-release cycle are modeled and calculated. Notably, it is impossible to calculate the capacitance directly because Miura-origami is a complex three-dimensional structure. Figure S2a–c shows the folding rule of the OPG with a single-generation unit. Surface 1 and Surface 6 are close to each other, while Surface 1 and Surface 2 are also close to each other through the other side of Surface 1. Therefore, the original structure can be divided into two capacitive units: the main capacitive unit corresponding to the complete parallelogram plane and the secondary capacitive unit corresponding to the triangular plane. Figure S2d–g shows the parameter settings for the parallelogram plane and triangular plane. Based on the origin of the capacitance equations, the capacitance of the main capacitive unit can be expressed as:

1 $${C}_{1}=\frac{\varepsilon a\,{\ln}(b\,\cos \,\theta )}{8k\pi \,\tan \,\theta }+{M}_{1}$$

where *ε* represents the dielectric constant of air; *k* represents the electrostatic force constant, *a* and *b* represent the width and height of the parallelogram electrodes, respectively; *θ* represents the included angle between the electrodes. The capacitance of the secondary capacitive unit can be expressed as:

2 $${C}_{2}=\frac{\varepsilon a}{8k\pi }\left[\frac{{\mathrm{ln}}(b\,\cos \theta )}{\tan \theta }-\frac{b\,\cos \theta -1}{b\,\sin \theta }\right]+{M}_{2}$$

where both *M*_1_ and *M*_2_ are constants. As shown in Figure S3, the capacitance of the zigzag-shaped TENGs (4 × 2 generation units) with 28 main capacitive units can be expressed as:

3 $${C}_{z}(\theta )=28\times \frac{\varepsilon a\,{\mathrm{ln}}(b\,\cos \,\theta )}{8k\pi \,\tan\, \theta }+{C}_{p}$$

where *C*_*p*_ represents the stray capacitance. The capacitance of the Miura-origami TENGs (4 × 2 generation units) with 7 main capacitive units and 45 secondary capacitive units can be further written as:

4 $${C}_{M}(\theta )=\frac{\varepsilon a}{8k\pi }\left[\frac{52\,{\mathrm{ln}}(b\,\cos \theta )}{\tan \theta }-\frac{45(b\,\cos \theta -1)}{b\,\sin \theta }\right]+{C}_{p}$$

When *b* cos*θ* = 1, *C*_1_ and *C*_2_ are all equal to zero. When *θ* and tan*θ* are equal to zero, *C*_1_ and *C*_2_ are invalid. When *a* is equal to 20 mm and *b* is equal to 17.32 mm, the capacitance variation Δ*C* can be estimated versus *θ* in the range of 0° to 86.69°. The capacitance variations of the main capacitive unit and the secondary capacitive unit can be calculated as 14.46 pF and 9.68 pF, respectively. Neglecting the minor stray capacitance *C*_*p*_, the capacitance variations Δ*C*_*z*_ and Δ*C*_*M*_ can be calculated as 404.88 pF and 536.92 pF in one compression cycle, respectively. In summary, the Miura structure exhibits more capacitance than the zigzag structure because it has a more compact structure, a twofold layer density and more secondary capacitor units.

The output performances of our OPGs (4 × 2 generation units) with three different generation modes are characterized by an electrodynamic shaker. The device is sandwiched between two parallel movable plates, and its displacement and compression force can be monitored in real time. Under the 24 mm original height, 8 mm excitation displacement and 3.0 g excitation acceleration, the time-domain output voltages for the OPGs in three different generation modes versus load resistance ranging from 10 MΩ to 110 MΩ are shown in Fig. 2a–c, respectively.

Figure 2d shows the voltage waveforms of the OPGs in three generation modes at a load resistance of 70 MΩ. The peak-to-peak voltages of the OPGs in zigzag, angle, and distance modes are 171 V, 260 V, and 324 V, respectively. Figure 2e shows the output powers of the OPGs in three generation modes versus load resistance ranging from 10 MΩ to 110 MΩ. Under the same vibration conditions, multimode OPGs with larger capacitance have lower capacitive reactance. The OPGs in the angle and distance modes have smaller internal resistances, therefore they also have smaller optimum load resistances. The maximum output powers of the OPGs in the zigzag, angle, and distance modes are 450 μW, 990 μW, and 1624 μW, respectively. The optimum output power of the OPG is increased by 3.6 times by changing the zigzag structure to the Miura structure. Figure S4 and Figure S5 show the Miura structure and zigzag structure with the same layer density. As shown in Fig. 2f, when the height of the origami structure is adjusted from 80 mm to 10 mm, it can be seen that the capacitance values of the OPGs in the angle and distance modes vary from 120 pF, 68 pF to 510 pF, and 478 pF, respectively. When the origami structure is adjusted from 80 mm to 5 mm, the capacitance values of the OPGs in zigzag mode vary from 46 pF to 360 pF. Because it is difficult to introduce the effect of the FEP electret layer in capacitance calculations, there is a discrepancy between the measured and calculated capacitance values. The predicted capacitance variation for the Miura structure is 32% (536.92 pF/404.88 pF) larger than that of the zigzag structure. The measured capacitance variation for the Miura structure is 30.5% (410 pF/314 pF) larger than that of the zigzag structure at the same layer density.

### Output performance with different excitation accelerations

**Fig. 3:**
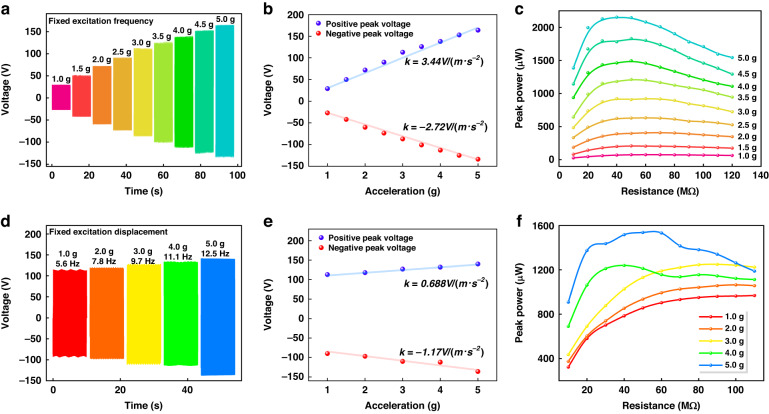
Output performance of the OPG with different excitation accelerations. **a** Output voltages with fixed excitation frequency and different excitation accelerations ranging from 1.0 g to 5.0 g under 50 MΩ; **b** voltage amplitude variations with excitation acceleration at fixed excitation frequency; **c** output powers with fixed excitation frequency and different excitation accelerations with varying load resistance ranging from 10 MΩ to 120 MΩ; **d** output voltages with fixed excitation displacements and different excitation accelerations ranging from 1.0 g to 5.0 g under 50 MΩ; **e** voltage amplitude variations with excitation acceleration at fixed excitation displacement; **f** output powers with fixed excitation displacements and different excitation accelerations with varying load resistance ranging from 10 MΩ to 110 MΩ

Figure 3a–c shows the output performances of the OPGs (4 × 2 generation units) for different excitation accelerations with a fixed excitation frequency of 15 Hz and an original height of 24 mm. Figure 3a, b shows the time-domain output voltages and amplitude variations of the OPGs with excitation accelerations ranging from 1.0 g to 5.0 g at a load resistance of 50 MΩ, respectively. The peak-to-peak output voltage increases from 61 V to 328 V when the acceleration changes from 1.0 g to 5.0 g. The positive peak voltage and negative peak voltage are quasi-linearly positively correlated with the excitation acceleration, based on slopes of 3.44 V/(m·s^−2^) and −2.72 V/(m·s^−2^), respectively. This result indicates that the OPG is expected to be used as a self-powered sensor with the function of measuring force and acceleration. Figure 3c shows the output powers for different excitation accelerations ranging from 1.0 g to 5.0 g with varying load resistances ranging from 10 MΩ to 120 MΩ. The optimum load resistance decreases with increasing acceleration. The maximum output power of 2152 μW is achieved at an acceleration of 5.0 g under the optimum load resistance of 50 MΩ, corresponding to peak power densities of 107.6 μW/cm^3^ and 213 μW/g.

Figure 3d–f shows the output performances of the OPGs (4 × 2 generation units) for different excitation accelerations with a fixed excitation displacement of 8 mm and an original height of 24 mm. Figure 3d, e shows the time-domain output voltages and amplitude variations of the OPGs with excitation accelerations ranging from 1.0 g to 5.0 g at a load resistance of 50 MΩ, respectively. The peak-to-peak output voltage increases from 207 V to 277 V when the acceleration changes from 1.0 g to 5.0 g. The positive peak voltage and negative peak voltage are quasi-linearly positively correlated with the excitation acceleration, with slopes of 0.688 V/(m·s^−2^) and −1.17 V/(m·s^−2^), respectively. This result shows that the OPGs with fixed excitation frequency have better acceleration sensitivity than the OPGs with fixed excitation displacements. Figure 3f shows the output powers for different excitation accelerations ranging from 1.0 g to 5.0 g with varying load resistances ranging from 10 MΩ to 110 MΩ. The optimum load resistance fluctuates when the acceleration increases from 1.0 g to 5.0 g. The maximum output power of 1537 μW is achieved at the optimum load resistance of 50 MΩ and an acceleration of 5.0 g.

### Output performance with different power generation units

**Fig. 4:**
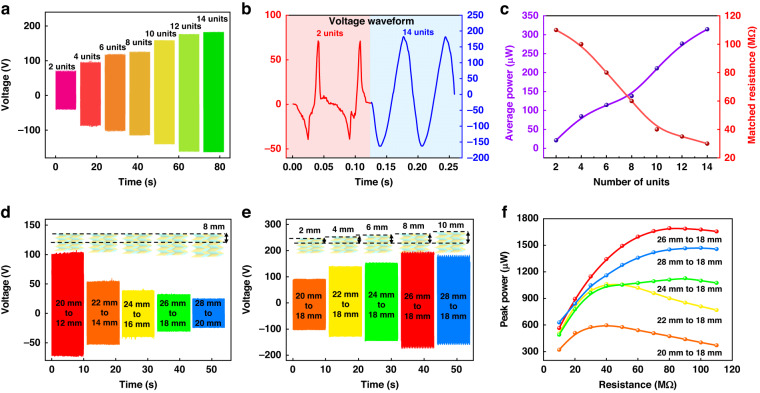
Output performance of the OPG versus the numbers of power generation units ranging from 2 to 14 and original heights ranging from 20 mm to 28 mm. **a** Output voltages with different numbers of power generation units; **b** voltage waveforms comparison between the OPGs with 2 generation units and 14 generation units at a load resistance of 50 MΩ; **c** output powers and optimum load resistances with different numbers of power generation units; **d** output voltages with different original heights; **e** output voltages with different excitation displacements; **f** output powers with different excitation displacements versus load resistance ranging from 10 MΩ to 110 MΩ

Figure 4a shows the time-domain output voltages of the OPGs with the numbers of power generation units ranging from 2 units (1 × 2 generation units) to 14 units (7 × 2 generation units) at an acceleration of 3.5 g and an original height of 24 mm. The voltage value linearly increases with increasing generation units. Figure 4b shows the comparison of the voltage waveforms between the 2 generation units and 14 generation units under 50 MΩ resistance. The peak-to-peak voltages of the OPGs with 2 generation units and 14 generation units are 112 V and 345 V, respectively. The waveforms of the OPGs with 14 generation units are more sinusoidal than those of the OPGs with 2 generation units, indicating that the output powers of the OPGs with more generation units have higher sustainability.

Figure 4c shows the output powers and optimum load resistances of the OPGs with power generation unit numbers in the range of 2 units to 14 units. With an increase in power generation units from 2 to 14, the output powers are significantly enhanced from 23 μW to 314 μW. This result demonstrates that the overall output powers of the OPGs have a significantly positive correlation with the number of power generation units. Fundamentally, the maximum output power can only be reached when the external load equals the internal impedance of the device. In general, the increase in power generation units is considered the parallel connection of multiple fewer power generation units, so the equivalent internal resistance of the OPG will be decreased with a growing number of power generation units. With an increase in power generation units from 2 to 14, the optimum load resistances are significantly reduced from 113 MΩ to 30 MΩ.

### Output performance with different original heights

Figure 4d–f shows the output performances of the OPGs (4 × 2 generation units) with different original heights under vertical pressing. Figure 4d shows the time-domain output voltages for the OPGs with different original heights ranging from 20 mm to 28 mm at 3.0 g excitation acceleration, 8 mm excitation displacements, and a load resistance of 50 MΩ. The peak voltage decreases when the original height increases from 20 mm to 28 mm. Figure 4e shows the time-domain output voltages of the OPGs with different excitation displacements ranging from 2 mm to 10 mm at 3.0 g acceleration, 18 mm compressed height, and a load resistance of 50 MΩ. The peak voltage increases before it decreases when the excitation displacement increases from 2 mm to 10 mm. The output performance mightily depends on the original heights and excitation displacements of the device. The highest output voltage can be obtained when the capacitance variation reaches the maximum value.

Figure 4f shows the output powers of the OPGs with different excitation displacements in the range of 2 mm to 10 mm versus load resistance in the range of 10 MΩ to 110 MΩ. When the device is deformed from the original height of 26 mm to the compressed height of 18 mm, the maximum output power of 1705 μW is achieved at the optimum load resistance of 80 MΩ. The optimum load resistance fluctuates when the excitation displacement increases.

### Output performance of the hexagonal electret generator

The output performance of the OPGs is significantly reduced because the surface charge density of the pre-charged FEP will decrease after long-term operation. Therefore, further investigations with different surface charge densities have been conducted. Figure S6 shows the voltage waveforms of the OPGs with different surface charge densities. When the surface charge density is increased from 185.45 μC/m^2^ to 741.8 μC/m^2^, the peak voltage changes from 60 V to 220 V. The OPG with a low surface charge density can also achieve an acceptable output performance. Furthermore, transmission lines are always affected by harsh weather conditions such as severe cold and sun exposure. Figure S7 shows the voltage waveforms of the OPGs at different ambient temperatures. When the devices are placed in −20 °C, 20 °C, 50 °C, and 70 °C environments, the peak voltages of the OPGs are 95 V, 190 V, 120 V, and 55 V, respectively. Moreover, the peak voltage recovers from 95 V to approximately 190 V after the OPG is removed from a −20 °C environment to room temperature. In summary, the OPG can stably operate for a long time in the harsh environments faced by transmission line systems.

To satisfy the need for a long-term and stable supply of electric energy in various walks of life, a large number of overhead transmission lines have been built. However, the conductors and arresters in overhead transmission lines are frequently exposed to intense vibration under accidental adverse meteorological conditions, which may lead to strand breakage, conductor breakage and other accidents. In this case, a hexagonal electret generator integrated with six-phase strip-shaped OPGs (2 × 4 generation units) is developed as “Buoy on Sky,” which is capable of harvesting vibration energy from various frequencies and amplitudes in random directions and monitoring the vibration conditions of transmission lines. Figure S8 shows the force-displacement curves of the OPGs with 2 × 4 generation units and 4 × 2 generation units. The stiffness *k* of the strip-shaped OPG (2 × 4 generation units) is measured as 335 N/m. Figure S9 shows the mass-spring model of the HEG, and the resonant frequency of the HEG is calculated as 9.2 Hz. We expect the HEG to obtain better output performance in the course of low-frequency vibration.

**Fig. 5:**
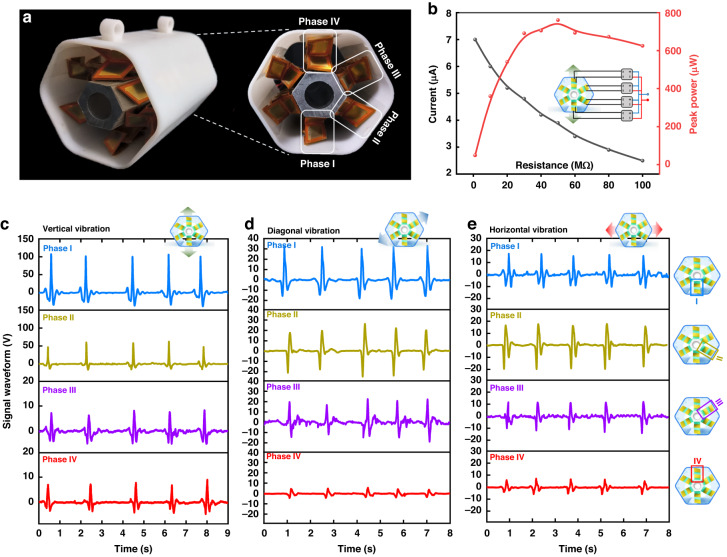
Output performance characterization of the HEG integrated with six-phase OPGs. **a** Photograph of the HEG and the side view of the OPGs located at phases I-IV; **b** output powers and currents of the HEG versus load resistance ranging from 1 MΩ to 100 MΩ; **c** signal waveforms generated from four-phase OPGs in the course of vertical vibration; **d** signal waveforms generated from four-phase OPGs in the course of diagonal vibration; **e** signal waveforms generated from four-phase OPGs in the course of horizontal vibration

Figure 5 shows the output performance characterization of the high-integrated HEG under different vibration directions. Figure 5a shows a photograph of the hexagonal electret generator integrated with six-phase OPGs (2 × 4 generation units) and a side view of the OPGs located at phases I-IV. The multiphase OPGs are plugged into the rectifier circuit and connected in parallel to characterize the output currents. Figure 5b shows the output powers and currents of the HEG in the course of vertical vibration versus load resistance ranging from 10 MΩ to 100 MΩ. The maximum output power of the HEG is 760 μW at the optimum load resistance of 50 MΩ.

To simulate the free vibration of the transmission line system, we excite the HEG every 2 s in different directions with an amplitude of 5 cm. The OPGs at phases I–IV are connected to a four-channel WIFI data acquisition card to characterize the voltage signal waveforms. Figure 5c–e shows the time-domain signal waveforms generated from four-phase OPGs when the HEG vibrates vertically, diagonally, and horizontally, respectively. During the vertical vibration, the peak voltages of the phase I-IV OPGs are 107 V, 52 V, 6.7 V, and 7.4 V, respectively. During the diagonal vibration, the peak voltages of the phase I-IV OPGs are 30 V, 14 V, 16.8 V, and 3.2 V, respectively. During the horizontal vibration, the peak voltages of the phase I-IV OPGs are 14.8 V, 15.6 V, 11.6 V, and 4.7 V, respectively. Due to the initial deformation caused by the central metal oscillator, a higher voltage signal can be generated from the OPG located at phase I during the all-direction vibration. In addition to the difference in the peak voltages, the signal waveforms and signal durations also contain information that can discriminate the vibration conditions.

### Application in AI-enabled transmission line vibration monitoring

**Fig. 6:**
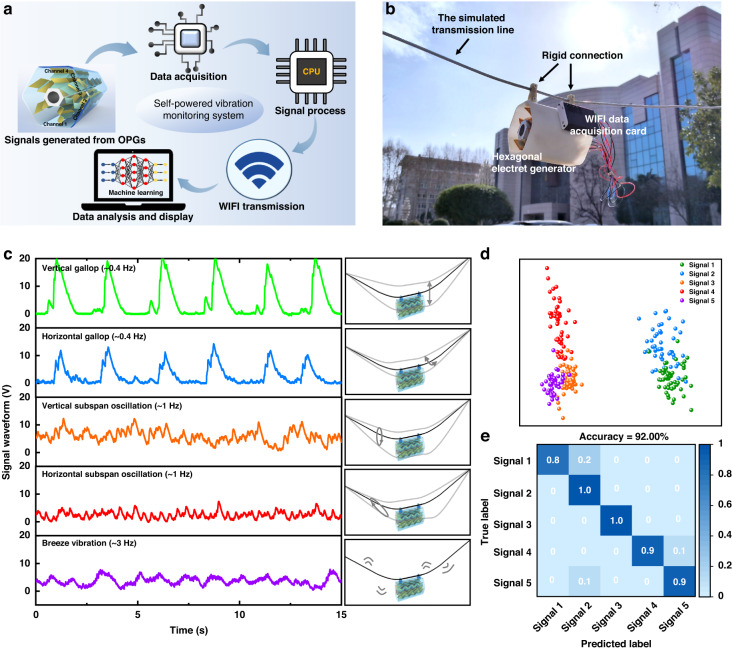
Vibration recognition enabled by self-powered sensing and machine learning. **a** Scheme diagram of the self-powered vibration monitoring system; **b** photograph of the HEG rigidly suspended on the simulated transmission line; **c** corresponding output signal waveforms generated from the HEG under five different vibration conditions; **d** PCA-LDA plot from five different vibration conditions; **e** confusion matrix for the recognition for five different vibration conditions

Based on the hexagonal electret generator previously proposed here, a self-powered monitoring system with the ability to recognize the vibration conditions of transmission lines is constructed. As shown in Fig. 6a, a distinctive voltage signal will be generated when the multiphase OPGs are integrated into the HEG and deformed with the vibrating of the simulated transmission lines. Then, the real-time signal waveforms are simultaneously collected by the front-end circuit and transmitted to the chip through the operational amplifier. Finally, the signals are wirelessly transmitted to the computer, and the real-time signals are displayed on the LabVIEW monitoring system and analyzed through machine learning algorithms. Figure 6b shows a photograph of the HEG integrated with six-phase OPGs rigidly suspended on the simulated transmission line platform and connected to a Wi-Fi data acquisition card.

According to the vibration frequency and amplitude, the vibration conditions of transmission lines are roughly divided into three types: high-frequency and micro-amplitude breeze vibration, medium-frequency and medium-amplitude sub span oscillation, and low-frequency and large-amplitude gallop. Breeze vibration can be considered a long-time vertical vibration caused by gentle winds. Subspan oscillation can be regarded as a vibration with an elliptical motion trajectory caused by a turbulent wind field. A gallop can be defined as a self-excited vibration caused by wind force on the transmission line. The graphical representations of these motions are shown in Fig. 6c. All these conditions threaten the safe operation of electrical power systems. With this platform, the six-phase OPGs in the HEG are plugged into the rectifier circuit and connected in parallel to collect the overall voltage signal waveforms that can characterize the vibration conditions.

Figure 6c shows the time-domain signal waveforms generated from the parallel OPGs under five different vibration conditions. When the transmission lines gallop vertically and horizontally at 50 mm amplitudes, a sawtooth signal waveform with 20 V peak voltage and 1.7 s duration (*Signal 1*) and a sawtooth signal waveform with 12 V peak voltage and 1.5 s duration (*Signal 2*) are collected, respectively. When the transmission lines oscillate vertically and horizontally at 30 mm amplitudes and 1 Hz frequency, a direct current (DC) signal waveform ranging from 3 V to 11 V (*Signal 3*) and a DC signal waveform ranging from 3 V to 5 V (*Signal 4*) are collected, respectively. Under breeze vibration, a linear DC signal waveform in the range of 1.5 V to 7 V (*Signal 5*) can be collected.

Although discrepancies between the signal waveforms under different vibration conditions are observed, it is almost impossible to manually extract enough features to distinguish them. To reveal the discriminative capacity of the HEG, as shown in Fig. 6d, high-dimensional sensory responses were projected into 2D space via principal component analysis (PCA) and linear discriminant analysis (LDA). The signals generated under different vibration conditions form distinctive clusters, demonstrating the feasibility of the HEG as a medium for transmission line vibration monitoring. Furthermore, the signal waveforms are analyzed and recognized by a one-dimensional convolutional neural network (1D-CNN) algorithm. The corresponding confusion map for vibration recognition is shown in Fig. 6e, which shows a high accuracy of 92%. This high recognition accuracy has further verified that the characteristics of various vibration conditions of transmission lines are distinct, although this phenomenon has rarely been investigated previously. In summary, these results suggest that the design of the multiphase electret generator is beneficial for continuous motion sensing, which is sensitive enough to recognize the characteristics of different vibration conditions.

## Conclusions

In this work, an innovative Miura-origami-inspired power generator constructed from one piece of electret thin film was developed, fabricated, and characterized. The Miura-origami fold is capable of transforming flat materials with a large surface area into simplified and compressed 3D structures with excellent stretchability, shape adaptability, and self-recovery characteristics. In addition, the two-sided corona discharge process has been employed to maximize the charge storage in FEP electrets and significantly enhance the contact triboelectrification (CE) mechanism and electrostatic induction mechanism. Moreover, the mechanical and electrical properties of the OPGs have been investigated and discussed comprehensively. At an acceleration of 5.0 g and frequency of 15 Hz, an instantaneous peak-to-peak voltage and optimum peak power of 328 V and 2152 μW were obtained, respectively, corresponding to peak power densities of 107.6 μW/cm^3^ and 213 μW/g.

Due to its excellent deformability, compact structure, and shape adjustability, our one-piece OPG has been successfully employed for VEH and IoT applications. For instance, a hexagonal electret generator integrating six-phase OPGs is developed as “Buoy on Sky,” which is capable of harvesting transmission line vibration energy from various frequencies and amplitudes in random directions, demonstrating its great potential in large-scale clean energy harvesting. Furthermore, vibration conditions of transmission lines can be recognized with 92% accuracy by analyzing the signal waveforms with machine learning algorithms. Overall, the outcomes of this work advance the potential to fuse origami art and energy harvesting techniques by demonstrating the viability of the OPG for various application scenarios.

## Materials and methods

Preparation of the OPG and HEG: The OPG prototype comprises a PCB board formed with 50 μm/100 μm/50 μm thick copper/LCP/copper sandwiched composite substrates, as well as two-sided 50 µm thick fluorinated ethylene propylene (FEP) films. The original PCB board is designed by Altium Designer Software. The HEG comprises six-phase OPGs, a hexagonal ABS outer shell designed by Solidworks Software and formed by 3D printing, and a hexagonal oscillator formed by computer numerical control (CNC) processing with 6061 aluminum alloy.

Characterization of the OPG and HEG: The measurement setup mainly consists of a function generator, a voltage amplifier, an electrodynamic shaker, and an accelerometer. The OPG is sandwiched between two parallel plates: one side is fixed to the shaker, and the other side is set to a positioning stage. An electrodynamic shaker with an amplitude range of +10 mm to −10 mm and a vibration acceleration range of 0 g to 10.0 g is used to simulate breeze vibration. A linear motor with an amplitude range of +100 mm to −100 mm and a vibration frequency range of 0.5 Hz to 5 Hz is used to simulate sub span oscillation and gallop. The electrical signal is recorded and displayed through an electrometer (Keithley 6514) and a data acquisition system (DAQ, NI USB-6289 M series, USA), which are connected and controlled by a computer. The capacitance of the OPG is measured using a precision LCR meter (Appellant AT811, CN). The charge distribution map is measured using a 3D potential scanner (Trek Model 347).

### Supplementary information

Supplemental Material
