# Supplementary material for: Constructing origami power generator from one piece of electret thin film and application in AI-enabled transmission line vibration monitoring
Source: Microsyst Nanoeng. 2023 Aug 7;9:101. doi: 10.1038/s41378-023-00572-6 (PMC10404589; doi:10.1038/s41378-023-00572-6)
Supplement: Supplementary file 1 — Supplemental Material [file 41378_2023_572_MOESM1_ESM.docx]

**Supplemental Material**

**Constructing origami power generator** **from one piece of electret thin film and** **application in AI-enabled transmission line vibration monitoring**

Boming Lyu^a,b^, Huipeng Zhou^a,b^, Yangyang Gao^a,b^, Xinhui Mao^a,b^, Fangzhi Li^a,b^, Jiyuan Zhang^a,b^, Dezhi Nie^a,b^, Wen Zeng^a^, Yonglin Lu^c^, Jin Wu^d*^, Zhaoshu Yang^e*^ and Kai Tao^a,b*^

^a^Ministry of Education Key Laboratory of Micro and Nano Systems for Aerospace, Northwestern Polytechnical University, Xi’an 710072, PR China

^b^Research & Development Institute of Northwestern Polytechnical University in Shenzhen, Shenzhen 518063, PR China

^c^Research Institute of State Grid Jiangsu Electric Power Co., Ltd., Nanjing 211103, PR China

^d^State Key Laboratory of Optoelectronic Materials and Technologies and the Guangdong Province Key Laboratory of Display Material and Technology, School of Electronics and Information Technology, Sun Yat-sen University, Guangzhou 510275, PR China

^e^National Key Laboratory of Human Factors Engineering, China Astronaut Research and Training Center, Beijing 100094, PR China


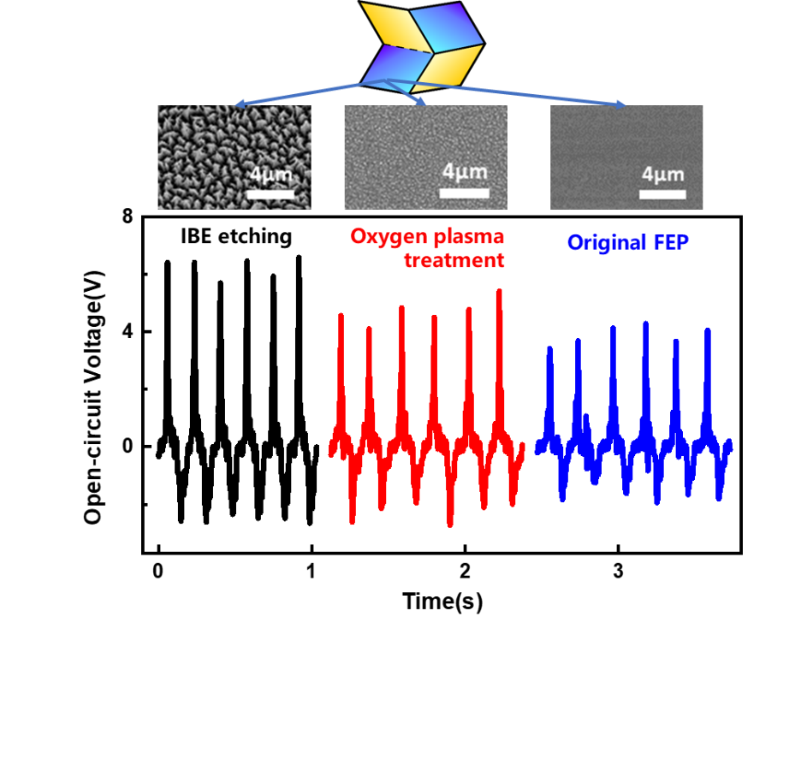


***Fig. S1:*** *Open-circuit voltage waveforms with different FEP surface microstructures.*

**
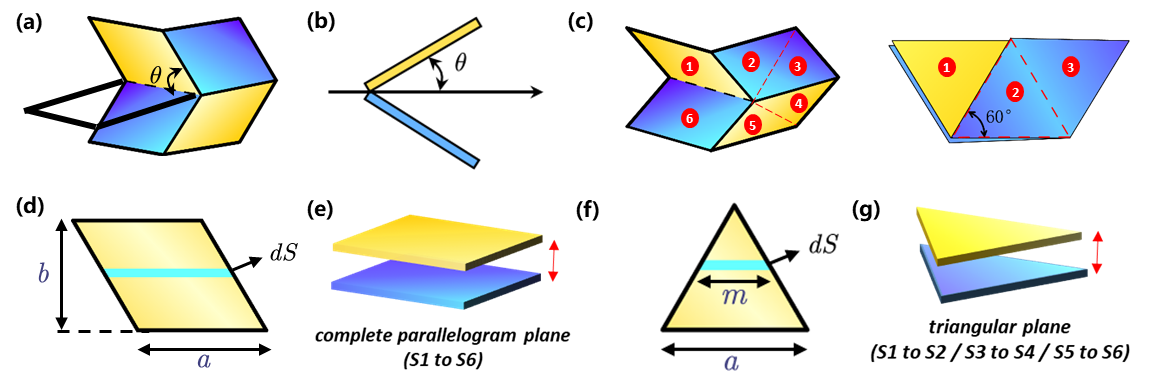
**

***Fig. S2.*** *(a-b) Schematic diagram of the angle θ between the two electrodes; (c) Schematic diagram of the folding rule of the OPG with single-generating unit; (d) The parameter settings of the main capacitive unit; (e) Schematic diagram of the main capacitive unit corresponding to the complete parallelogram plane; (f) The parameter settings of the secondary capacitive unit; (g) Schematic diagram of the secondary capacitive unit corresponding to the triangular plane.*

**Figs. S2a-b** show the angle between the two electrodes. **Fig. S2c** shows the folding rule of the OPG with single-generation unit. Surface 1 and surface 6 are close to each other, while surface 1 and surface 2 are also close to each other through the other side of surface 1. Therefore, the original structure can be divided into two capacitive units: the main capacitive unit corresponding to the complete parallelogram plane (surface 1 to surface 6) and the secondary capacitive unit corresponding to the triangular plane (surface 1 to surface 2, surface 3 to surface 4 and surface 5 to surface 6 on the back). The dimensions of the main capacitive unit have been shown in **Figs. S2d-e**, where the width and height of the electrodes are “” and “”, the integration region is “”.

For the main capacitive unit, the distance between the two electrodes can be expressed as: and the integration region can be expressed as: . The capacitance of the main capacitive unit corresponding to the complete parallelogram plane can be expressed as:

(2-1)

(2-2)

(2-3)

(2-4)

For the secondary capacitive unit, the distance between the two electrodes can be expressed as: and the integration region can be expressed as: . The capacitance of the secondary capacitive unit corresponding to the triangular plane can be expressed as:

(2-5)

(2-6)

(2-7)

(2-8)

Both and are constants. When , and are all equal to zero. When and are equal to zero, and are invalid. So the angle ranges from 0.001° to 86.69°. The air dielectric constant is 1.0 and the electrostatic force constant is 8.988×10^9^ N·m^2^/C^2^. The width and height of the electrodes are 20 mm and 17.32 mm, respectively. Take these parameters in the equations (2-4) and (2-8). The capacitance variations of the main capacitive unit and the secondary capacitive unit can be calculated as 14.46 pF and 9.68 pF, respectively.


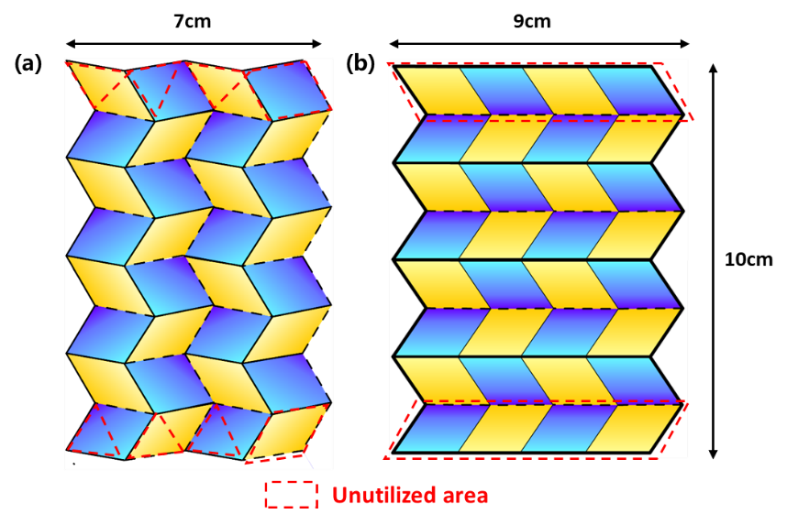


***Fig. S3:*** *Comparison of the Miura structure and zigzag structure.*

For the Miura-origami TENG (4×2 generation units) with 7 main capacitive units and 45 secondary capacitive units, the capacitance can be further written as:

(3-1)

For the conventional zigzag-shaped TENG (4×2 generation units) with 28 main capacitive units, the capacitance can be expressed as:

(3-2)

Ignoring the minor stray capacitance , the and can be calculated as 536.92 pF and 404.88 pF in one compress cycle, respectively. In summary, the reason of why the Miura structure has more capacitance and performs better than the zigzag structure is as follows:

1. The secondary capacitive unit only occupies half of the area of the main capacitor unit, but the capacitance variation is much higher than half of . And the Miura structure has more secondary capacitive units.
2. Compared to the zigzag structure, the Miura structure has more effective power generation units (7+45/2=29.5 units>28 units), as shown in **Figs. S3**.
3. Miura structure is more compact and has higher layer density than zigzag structure (=10 cm×7 cm×1.732 cm＜=10 cm×9 cm×1.732 cm).


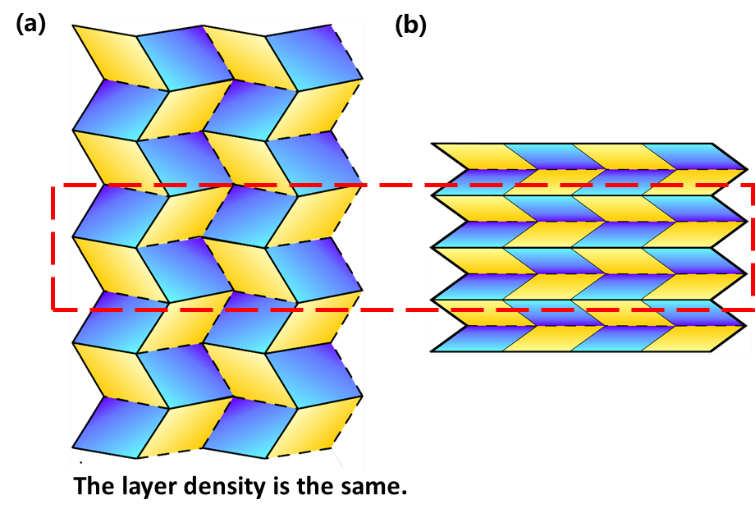


***Fig. S4:*** *The Miura structure and zigzag structure with the same layer density.*


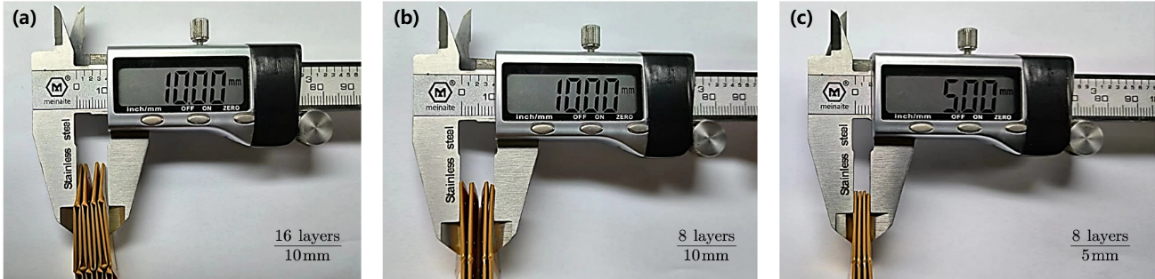


***Fig. S5:*** *(a) The Miura structure compressed at 10 mm; (b-c) The zigzag structures compressed at 10 mm and 5 mm, respectively.*


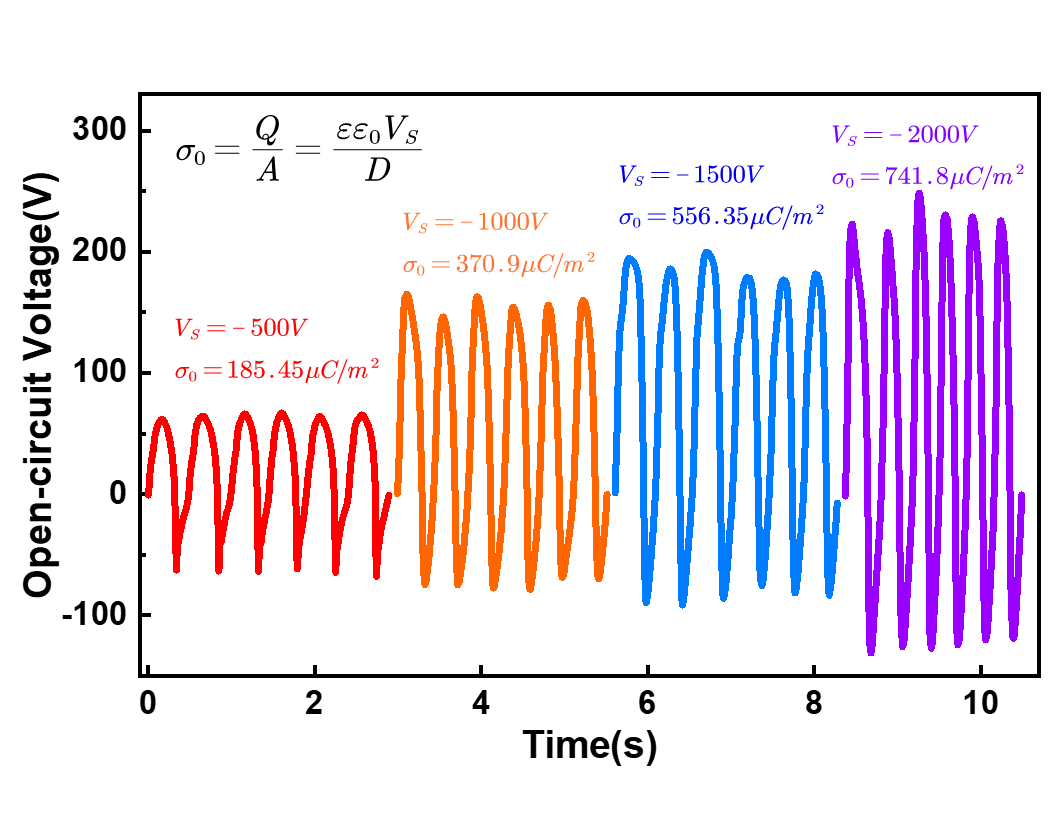


***Fig. S6:*** *Open-circuit voltage waveforms with different surface potentials and charge densities.*


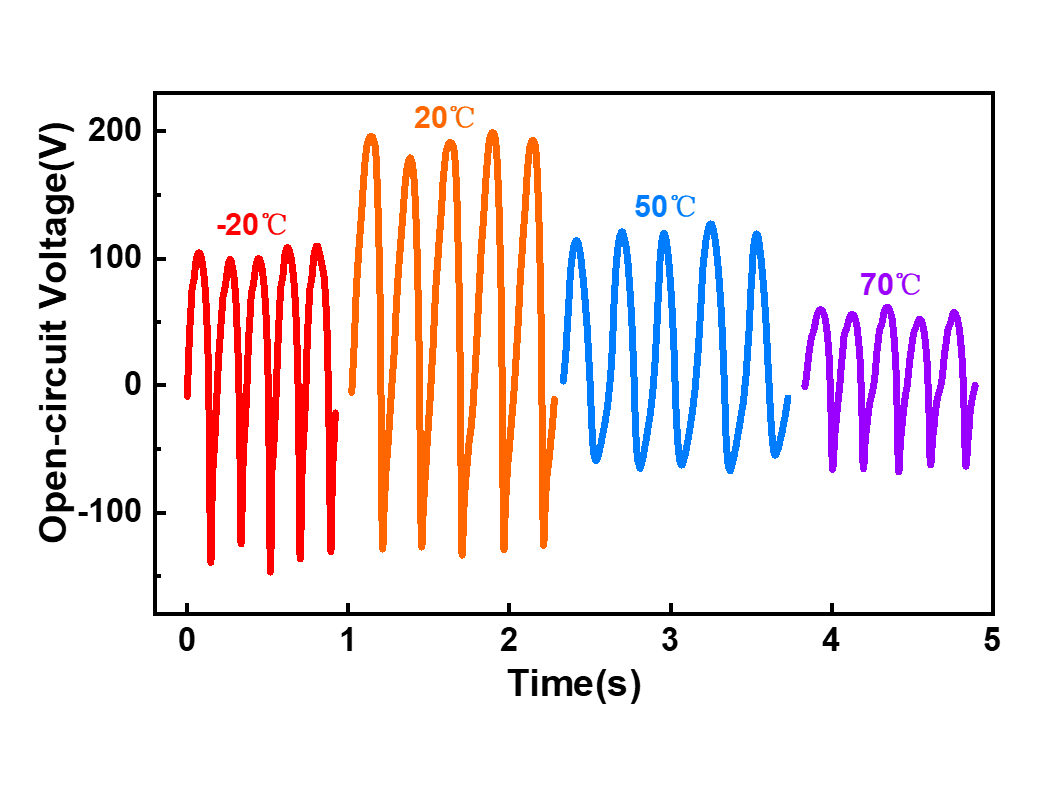


***Fig. S7:*** *Open-circuit voltage waveforms with different ambient temperatures.*


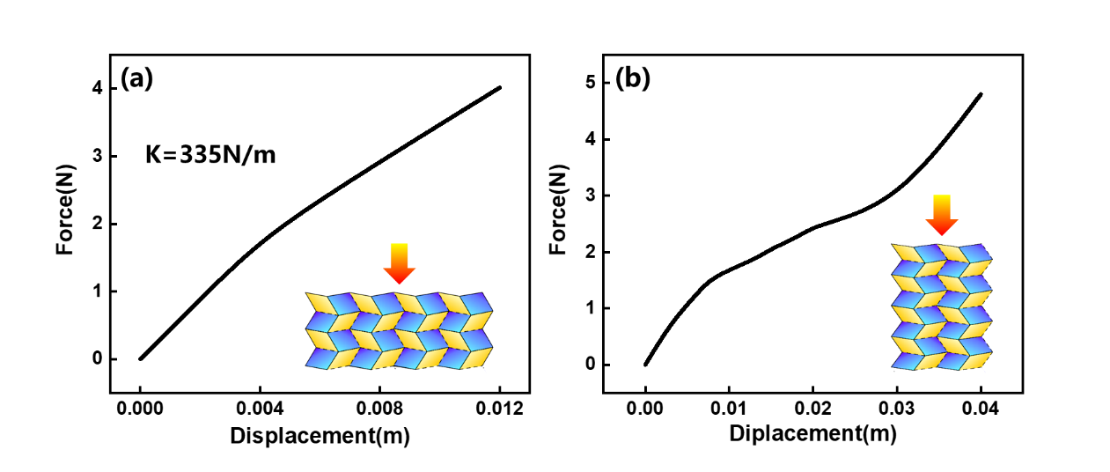


***Fig. S8:*** *(a) The force-displacement curve of the OPGs (2×4 generation units); (b) The force-displacement curve of the OPGs (4×2 generation units).*

In our work, a hexagonal electret generator (HEG) integrated with six-phase strip-shaped OPGs is developed, capable of harvesting vibration energy from various frequencies and amplitudes in random directions and monitoring the vibration conditions of transmission lines. We have measured and plotted the force-displacement curves of the OPGs with 2×4 generation units and 4×2 generation units, respectively. As shown in **Fig. S8**, the stiffness of the strip-shaped OPG (2×4 generation units) is measured as 335 N/m.

Based on the Energy Method and Conservation law of kinetic and potential energy:

(8-1)

, (8-2)

, (8-3)

So (8-4)

For sinusoidal vibration:

(8-5)

(8-6)


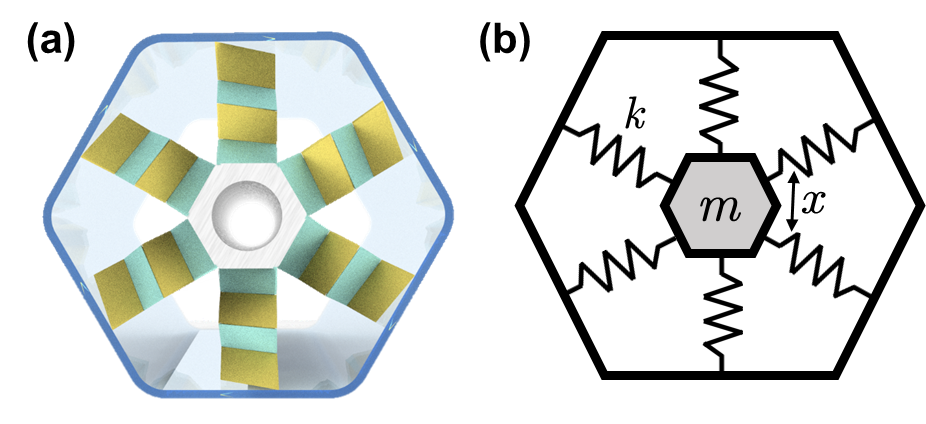


***Fig. S9:*** *(a) Schematic diagram of the HEG; (b) Mass-spring model of the HEG.*

As shown in **Fig. S9**, when the central oscillator moves in the x-axis direction, the potential energy of the HEG system can be expressed as:

(9-1)

(9-2)

The equivalent stiffness and natural angular frequency can be expressed as:

(9-3)

(9-4)

And the resonant frequency of the HEG can be calculated as:

(9-5)
